# Bacterial community composition in costal dunes of the Mediterranean along a gradient from the sea shore to the inland

**DOI:** 10.1038/srep40266

**Published:** 2017-01-11

**Authors:** Haggai Wasserstrom, Susanne Kublik, Rachel Wasserstrom, Stefanie Schulz, Michael Schloter, Yosef Steinberger

**Affiliations:** 1 The Mina and Everard Goodman Faculty of Life Sciences, Bar-Ilan University, Ramat-Gan 5290002, Israel; 2 Research Unit for Environmental Genomics, Helmholtz Zentrum München, German Research Center for Environmental Health, 85764 Neuherberg, Germany

## Abstract

Sand dunes are unique ecosystems with distinct features which limited the accumulation of biomass. The distance from seashore affects both the physical properties of the sand dunes and the biota living above- and below ground. The goal of the present study was to determine the effects of the distance from shore to inland on soil bacterial community composition during wet and dry season. We studied a chronosequence of sites close to the eastern Mediterranean coast. Bacterial diversity was assessed using directly extracted DNA from soil samples and 16 S ribosomal RNA gene fingerprinting. Our data indicates a significant influence of season and site on bacterial community structure. We showed that during the wet season soil organic matter, pH and salinity strongly influence bacterial community composition, whereas during the dry period bacterial diversity was mainly driven by the shortage of water at all sites. Consequently diversity was lowest during dry season at dunes close to the shore, whereas during the wet season the higher water content and the reduced salinity at the dunes which are more at the inland induced an increase in diversity, which illustrates the pronounced dynamics of microbial communities in soil over a season mainly at inland dunes.

Sand dunes are widely distributed across the globe, covering 6 × 10^6 ^ km^2^ of its land surface^1^. In contrast to the relatively stable nature of soil, sand dune ecosystems are dynamic and are mostly in a state of successional change^2^,^3^. They represent an ecosystem with strong gradients of physical stress which severely limit above and below ground biomass accumulation.

Coastal dunes are additionally characterized by the impact of the marine ecosystem, including tidal rhythms, climatic conditions and sedimentary deposition^4^. Thus typically a distinct gradient across a coastal dune-field is formed from the shore to the inland, with the typical sandy character as a common property, but differences in other abiotic and biotic factors along the transect^5^. Many studies have demonstrated the influence of these strong abiotic gradients on plant community composition and distribution^6^,^7^,^8^ as well as on macrofauna^9^,^10^,^11^.

However, information on the effects on soil microbial communities is limited and restricted to cold and wet environments^12^,^13^. Microorganisms are a fundamental component of any terrestrial ecosystem due to their key role in organic matter decomposition, nutrient cycling, and the development of soil structure^14^, especially in sand dune environments^15^. Changes in soil microbial community abundance and composition, can significantly affect the dynamics of these essential processes which affect also above-ground biodiversity. Moreover, the activity of the soil-sand microflora may influence the maturation and stabilization of sand dune ecosystems^16^,^17^ by steering the aggregation of sand dunes by secretion of polysaccharides and other compounds resulting in an adhesion of sand particles into larger aggregates, eventually^18^,^19^.

Thus the aim of this study was to investigate the differences in bacterial diversity across a sand dune ecosystem gradient, from the sea to the inland, in the eastern Mediterranean coast. Soil samples were collected during the dry season (summer) and wet season (winter) from the study area, consisting of shifting and semi-stabilized coastal sand dunes, at six sites along a 4 km transect, from the sea to inland. Bacterial communities were analyzed using molecular fingerprinting approaches based directly extracted DNA from the samples and subsequent PCR amplification of 16 S rRNA genes. The obtained data was correlated to abiotic soil properties.

## Results and Discussion

### Soil abiotic parameters

Abiotic soil properties are summarized in Table 1 and Table 2. The mean soil moisture in the different soil samples ranged from 2.13 to 3.63% in the wet season and from 0.13 to 0.21% in the dry season. No differences were observed between the six sampling sites when comparing samples from the dry- respectively wet season, with the exception of D_4_, where significantly lower soil moisture content was measured in the wet season compared to the other sites. Organic matter content was low in all soil samples, ranging from 0.01 to 0.17%. The highest amounts of soil organic matter were found during the wet season at site D_5_. For the sites with the highest values for organic matter content during the wet season (D_3_ and D_5_) values dropped significantly for the dry season. For the other sites no clear differences between both sampling time points were observed. Soil salinity in the dry season, ranged between 1.90 to 3.20 mS/cm, compared to the wet season with values between 2.04 and 2.92 mS/cm. During the dry- and wet season a clear decrease of salinity was observed along the gradient towards inland. The soil pH was slightly alkaline at both time points of sampling. Whereas a slight decrease in soil pH could be observed from the shore to the inland during the dry season, no differences were obtained during the wet season.

### The effect of distance from seashore on bacterial community composition

**Figure 1:**
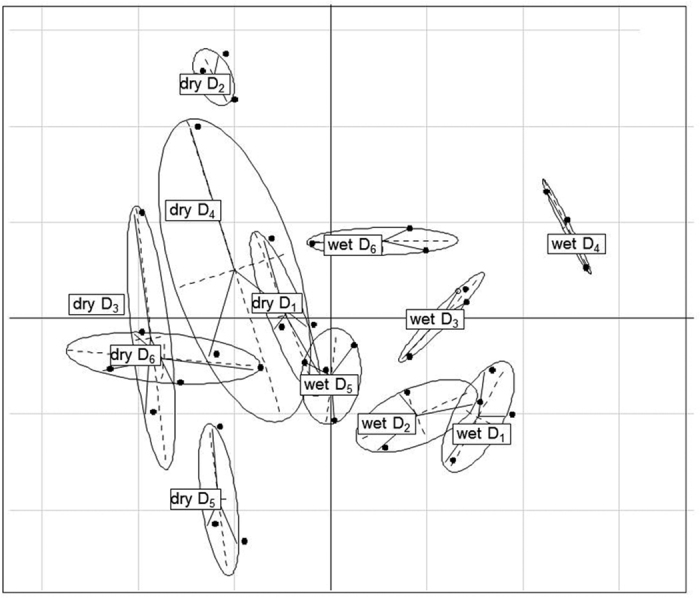
Between-group analysis (BGA), based on principal component analysis of Hellinger transformed T-RFLP dataset for 16 S rRNA gene fragments from the different soil sample obtained from the six dunes across the transect (D_1_ - 100 m; D_2_ - 200 m; D_3_ - 400 m; D_4_ - 1000 m; D_5_ - 2500 m; and D_6_ - 4100 m) during wet and dry season (n = 3).

Bacterial ß-diversity varied between sampled sites and season as shown by permutation-based multivariate analysis of variance (Table 3). The significance of seasonality, i.e., wet and dry seasons, for the composition of soil bacterial communities has been described also for other sandy and arid environments like deserts^20^,^21^. The between group analysis of T-RFLP pattern confirmed the significant effect of the season on the soil bacterial community composition (*p* < 0.001, Fig. 1). Mainly during the wet season a clear clustering of the different sites was visible. Only the sites D_1_ and D_2_, which are closely located to the shore showed some overlap. During the dry season interestingly also D_1_ and D_2_ differed in their bacterial community composition. However an overlap between D_1_, D_3_, D_4_ and D_6_ was visible, which was mainly related to the pronounced heterogeneity of the replicates of D_4_. At this site strong growth of algae and cyanobacteria has been observed during the wet season, which resulted also in the formation of the most distinct cluster in winter. Obviously the formation of soil crusts during the wet season induced a strong heterogeneity in the soils in the dry season, which has been also described in other studies^22^.

**Figure 2:**
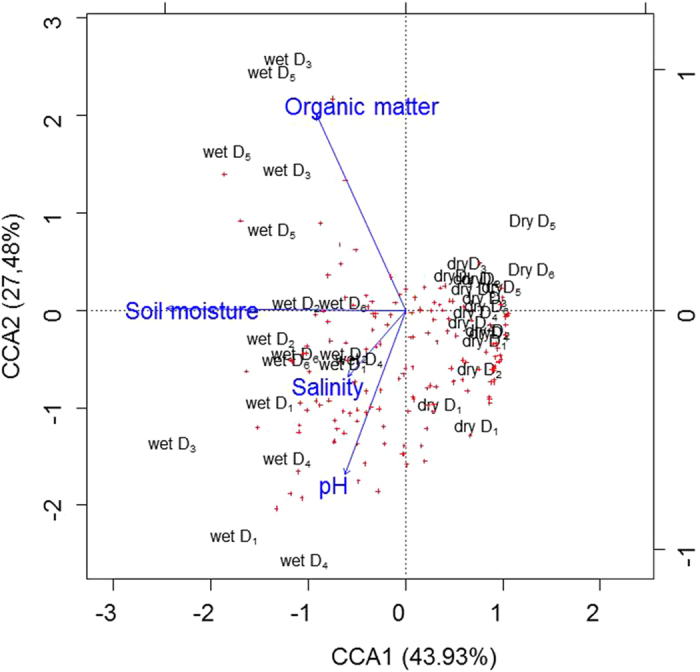
Canonical correspondence analysis (CCA) of T-RFs with respect to the abiotic parameters. Samples are represented by labels indicated season followed by a number indicating the sampled location along the transect. T-RFs are represented by crosses and abiotic parameters are indicated by arrows (n = 3).

As shown by CCA, bacterial communities were mostly influenced by the differing soil moisture at the two sampling time points, which induced a clear separation between bacterial communities, independent from the sampling site (Fig. 2). As temperature also strongly differs between the two sampling time points, with lower temperatures in January, the time point where higher soil moisture levels were measured, the observed effect of soil moisture on bacterial communities might be considered even higher under constant air- and soil temperature, as the reduced temperature in January might reduce the growth of some bacterial taxa despite the presence of higher water contents in soil. Whereas during the dry season, no additional abiotic drivers could be identified, during the wet season, where water was not the main limiting factor for bacterial growth a clear link between soil organic matter content and bacterial community composition was observed for samples obtained from D_3_ and D_5_. In contrast bacterial communities which developed on dunes closer to the sea side during the wet season (D_1_ and D_2_) were more driven by salinity and soil pH. These results are in line with previous studies^9^, where phospholipid fatty acids were used as marker for the analysis of microbial communities and dunes along a shorter transect from the shore to the inland were studied. Here the authors described a clear link between the increase of vegetation and microbial community structure at the dunes from the inland.

In total, 95 bacterial T-RFs have been identified. In accordance with our expectations lowest α− diversity (measured as the number of T-RFs per sample; Table 4) was found at the site closest to the sea (D_1_) during the dry season. Here obviously the harsh conditions present only allowed a survival of selected strongly adapted bacteria. Interestingly as a result of increased water availability, during the wet season, diversity increased significantly and was comparable to most other sites. In contrast to our expectations mainly during the dry period in summer diversity at D_4_ was reduced. The reasons for this observation need to be investigated in more detail in the future. α-diversity at the other sites was comparable and did not change significantly during wet and dry season at site D_2_.

**Figure 3:**
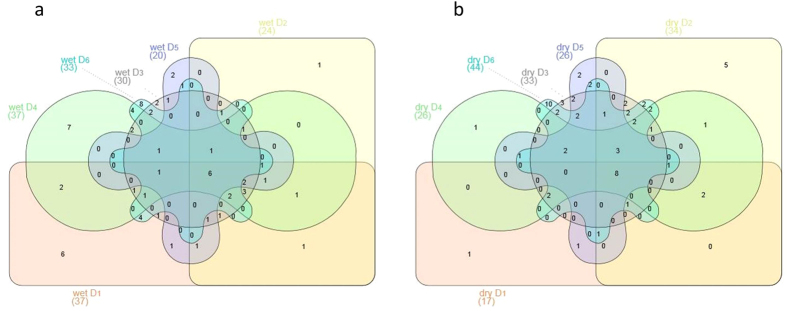
Definition of a core microbiome: Based on t-RFLP data (16 S rRNA) of soil samples from the six different dunes a VENN diagram was calculated for the dry season (**a**) and the wet season (**b**). Shared OTUs representing the core microbiome as well as uniquely found OTUs are shown (n = 3).

Based on the obtained data a Venn diagram has been calculated, which allowed the definition of a core microbiome for all samples (Fig. 3). As a result of the low diversity of T-RFs at D_1_ during the dry season, the number of shared T-RFs between all samples is quite low in absolute numbers (6 out of 95). In relative numbers about 30% of all T-RFs occurring at site D_1_ are also present at the other sites. During the wet season this situation changed and much more “site specific” T-RFs were visible, as only 21% of the T-RFs present at site D_1_ could be also detected at the other sites. However it needs to be taken into account that here, in contrast to data sets where sequencing data has been used, the definition of an OTU is based on peak distribution pattern, which is only an indirect measure for sequence heterogeneity, although T-RFLP based analysis of amplified 16 S rRNA gene fragments has been shown to be comparable in resolution of operational taxonomic units compared to sequencing based approaches mainly when TRFLP based OTUs were compared to the OTU_90_ level (which reflects the level of bacterial families) assessed by sequencing^23^.

Overall, the differences between the bacterial communities were greater during the wet season compared to the dry one. This can be attributed to the combined effect of few factors. During the wet season there was a significant increase in soil moisture which is the main environmental factor influencing the soil microbial community^24^,^25^. Especially in arid environments despite the lower temperature, the increase in water content provides better conditions for activity and development of microbial communities^26^. The connection of microbial biomass and soil moisture was also demonstrated in a sand dune forest ecosystem^27^. Moreover, during the wet season there are significantly strong winds in the Israeli Mediterranean cost^28^ which affect sand movement. This movement of sand by wind had been shown to affect fungi and nematodes in coastal sand dunes^29^. This effect changes with the distance from the shore and the topography and therefore these observations are more visible close to the shore than at inland sites.

Our data clearly demonstrates the dynamics of microbial communities in the dunes in different seasons depending on their location. However the use of the here presented molecular fingerprinting approach to assess bacterial community structure, does not allow to draw valid conclusions on the phylogeny of the related microorganisms. Thus future studies are needed where modern sequencing approaches are implemented to understand which groups which are mostly affected by water shortage and salinity. Furthermore an approach addressing the questions on the mechanisms how microbes can adapt to the harsh conditions present mainly during summer at the dunes close to the seaside using a combined approach of metagenomics assessment and isolation would be of interest also for a possible biotechnological application of the related bacteria in the future.

## Methods

### Study site and sampling

The study was conducted at the coastal sand dunes located in the northern Sharon Plains near Caesarea, Israel (32°48′N and between 34°88′E and 34°93′E), along a 4 km transect, from the sea to inland. The climate is sub-humid Mediterranean with a multiannual mean rainfall of 580 mm, falling mainly during winter–early spring (October to March), with maximum rainfall in December. The mean minimum daily temperature reaches 10.5 °C in January (wet season), while the mean maximum temperature reaches 28.5 °C in August (dry season). During the dry summer season the wind regime is stable, whereas winter winds are intense. The sandy dunes at the study site vary from shifting sand to semi- and fully stabilized dunes with a vegetation cover dominated by shrubs and herbaceous perennials. The color of the sand varies from yellowish (closer to shore) to yellow-reddish (with distance from the shore), according to the amount of fine-grained (silt and clay) deposits and organic matter^30^.

Soil samples were collected from 0–10 cm depth, at six locations (n = 3) along a sand dunes transect at 100 m (-D_1_), 200 m (-D_2_), 400 m (-D_3_), 1000 m (-D_4_), 2500 m (-D_5_), and 4100 m (-D_6_) distance from the shore. Soil samples were collected from plant free locations on January 30 (wet season) and August 21 (dry season), 2014, in order to avoid the influence of different plants creating different microhabitats and soil microbial communities^20^,^31^,^32^.

Air temperature at the days of sampling was 17.2 °C in January respectively 27.9 °C in August. Soil temperature ranged from 15.9 °C (January) to 27.1 °C (August). No significant differences were observed at the two dates, neither for air- nor for soil temperature, for the different sampling sites (D_1_ – D_6_). Precipitation in the week prior to sampling was 2.4 mm (January) respectively 0.0 mm (August).

A total of 36 soil samples were collected during the study period. After sampling, soil samples were immediately placed in individual polyethylene bags and transported to the laboratory in an insulated cooler. Stones, roots, and other organic debris were removed from, prior to physicochemical and biological analyses; samples were stored at −20 °C for biological respectively 4 °C for physical analysis.

### Soil abiotic parameters analysis

Samples from each replicate were analyzed for soil moisture (SM), soil organic matter (OM), salinity and pH, as follows: SM was determined gravimetrically by drying the soil samples in an oven at 105 °C for 48 h and measuring the mass loss; OM was analyzed by oxidation with dichromate in the presence of H_2_SO_4_^33^; salinity was measured by electrical conductivity in a soil-water suspension (soil: water 1:10) using an autoranging EC/temp meter (TH2400, EI-Hamma); the pH was determined in the filtered supernatant of a soil slurry (soil: water 1:2) using a combined pH electrode in the of a mixture of soil and tap water (1:2).

### Soil microbial community analysis

Genomic DNA was extracted from each sample using the GeneAll^®^ Exgene^TM^ Soil DNA mini kit according to the manufacturer’s instructions (GeneAll Biotechnology, Seoul, Korea).

The bacterial 16 S rRNA gene was amplified from the total DNA using FAM (6-carboxyfluorescein)-labeled polymerase chain reaction (PCR) forward primer and an unlabeled reverse primer. The primers used for the 16 S rRNA gene were B27-FAM (5′-AGAGTTTGATCCTGGCTCAG)^34^ and 1401-R (5′- CGGTGTGTACAAGACCC)^35^, respectively. For the 16 S rRNA gene, the 50 μl reaction mixture contained 5 μl of 10x PCR buffer, 2.5 μl MgCl2 50 mM, 5 μl dNTPs 2 mM, 1 μl of each primer, 1.25 μl DMSO, 1 μl BSA 3%, 500 U Taq-Polymerase, 30.75 μl H2O (DEPC), and 2 μl DNA from each sample. Amplification of the 16 S rRNA gene was performed in the T3 Thermocycler (Biometra, Germany) using the following program: a 5 min hot start at 94 °C, followed by 30 cycles consisting of denaturation (1 min at 94 °C), annealing (1 min at 57 °C), extension (1 min at 72 °C), and a final extension step for 10 min at 72 °C.

### T-RFLP analysis

Diversity analysis by terminal restriction fragment length polymorphism (t-RFLP) was performed targeting the bacterial 16 S rRNA gene. For amplification, primer pairs and PCR profiles were performed, as described for PCR. Purification of the PCR products was performed with the NucleoSpin Gel and PCR clean-up kit (Macherey-Nagel, Germany), and digestion was performed using the restriction enzyme MSP1 (New England Biolabs, Germany) for the 16 S rRNA gene. For digestion with MSP1, 10 U of the enzyme, 1 μl of 10x buffer, x μl H2O (DEPC), and 200 ng of the amplicon (final volume 10 μl) were used by using following program on the thermocycler: 4 h at 37 °C and 20 min at 80 °C. The digested amplicons (50 ng) were desalted and purified with the Gel and PCR clean-up kit (Macherey-Nagel). One microliter was then mixed with 13 μl of Hi-Di formamide (Applied Biosystems, Germany) containing a 400-fold dilution of a 6-carboxy-X-rhodamine-labeled MapMarker 1000 ladder (Bio-Ventures, USA), denatured (5 min at 95 °C), cooled on ice, and size-separated on a 3730 DNA analyzer (Applied Biosystems). Electrophoresis was performed with POP-7 polymer in a 50-cm capillary array under the following conditions: 10 s injection time, 2 kV injection voltage, 7 kV run voltage, 66 °C run temperature, and 63 min analysis time.

Analysis of electropherograms was performed using the software PeakScanner 2 (Life Technologies, USA). Fragments shorter than 50 bp were omitted before datasets were further processed using T-REX software ((http://trex.biopc.org)^36^. For noise filtering the “Std dev multiplier for fluor B” was set to 0.8 using peak height. Operational taxonomic units (OTUs) were defined as peaks within a clustering threshold of 1 bp. Statistical evaluation of T-RFLP data was performed by applying permutational multivariate analysis of variance by using the Adonis function in the R environment. The data set was Hellinger transformed^37^.

### Data analysis

Statistical analysis was implemented using the R environment (version 3.2.0) (R Core Team, 2015). The impact of the soil type abiotic soil properties on 16 S rRNA gene abundance of 16 S rRNA gene (logarithmic) was tested by analysis of variance (ANOVA). Here normal distribution was verified using the “Shapiro-Wilk normality test” in R), while the effect of soil typeabiotic soil properties on diversity of 16 S rRNA genes diversity was investigated by permutational multivariate analysis of variance using distance matrices. For determination of a core microbiome VENN diagrams (http://bioinformatics.psb.ugent.be/webtools/Venn/) were calculated based on filtered t-RFLP data (fragments occurring in less than 2 biological replicates were omitted)^38^.

To analyze regression-coefficients and correlations between two data matrices Pearson correlation was applied Canonical correspondence analysis (CCA) (regression based) and regularized canonical correlation analysis (RCC) (correlation based) were applied for soil parameter compared with 16 S rRNA gene diversity data (t-RFLP). For CCA and RCC the t-RFLP data was normalized using an Anscombe transformation to stabilize the variance followed by a square root transformation of the relative abundances.

## Additional Information

**How to cite this article**: Wasserstrom, H. *et al*. Bacterial community composition in costal dunes of the Mediterranean along a gradient from the sea shore to the inland. *Sci. Rep.* **7**, 40266; doi: 10.1038/srep40266 (2017).

**Publisher's note:** Springer Nature remains neutral with regard to jurisdictional claims in published maps and institutional affiliations.

## Figures and Tables

**Table 1:** Changes in the mean values of abiotic parameters in soil samples collected from the six locations (D_1-6_), during the wet and dry seasons.

	Soil moisture (%)		Organic matter (%)		Salinity (mS/cm)		pH	
	Wet	Dry	Wet	Dry	Wet	Dry	Wet	Dry
D_1_	3.01^a^	0.21^a^	0.025^a^	0.026^a^	2.92^a^	3.20^a^	7.67^a^	7.78^a^
D_2_	3.72^a^	0.17^a^	0.061^b^	0.031^a^	2.24^b^	2.41^b^	7.80^a^	7.74^a^
D_3_	3.63^a^	0.17^a^	0.118^c^	0.028^a^	2.18^b^	2.01^c^	7.66^a^	7.62^b^
D_4_	2.13^b^	0.13^a^	0.016^a^	0.029^a^	2.04^c^	2.11^c^	8.05^a^	7.55^b^
D_5_	3.63^a^	0.17^a^	0.170^d^	0.046^b^	2.35^b^	1.90^c^	7.61^a^	7.59^b^
D_6_	3.10^a^	0.17^a^	0.016^a^	0.035^a^	2.28^b^	2.35^b^	7.86^a^	7.70^a^

The letters represent statistically significant differences (*p* < 0.05) between the six locations during the same season.

**Table 2:** Univariate analysis of variance for abiotic soil parameters with seasons and locations.

	Season		Location		Season × Location	
	*F-test*	*p-value*	*F-test*	*p-value*	*F-test*	*p-value*
Soil moisture	780.29	<0.0001	4.19	0.0021	3.95	0.0032
Organic matter	8.76	0.0042	6.8	<0.0001	4.05	0.0027
Salinity	0.03	NS	24.62	<0.0001	4.31	0.0017
pH	13.41	0.0005	2.73	0.0258	0.96	NS

**Table 3:** Permutation-based multivariate analysis of variance of 36 T-RFLP profiles of the two seasons at 6 different locations based on Yue-Clayton distance matrix.

	Df	SS	MS	*F*-test	*p*-value
**Season**	1	1.662	1.66199	4.6623	<0.001
**Location**	5	3.645	0.72899	2.045	<0.001
**Season × Location**	5	2.9359	0.58718	1.6472	<0.001
**Residuals**	24	8.5554	0.35647		
**Total**	35	16.7982			

Df-degrees of freedom; SS-sum of squares; MS-mean of squares.

**Table 4:** Bacterial diversity as assessed by T-RFLP of the 16 S rRNA gene.

	Wet	Dry
	Mean	Mean
D1	37.00^a^	18.33^a^
D2	30.33^a^	36.67^b^
D3	33.33^a^	38.33^b^
D4	38.67^a^	30.67^b^
D5	23.00^b^	27.67^c^
D6	33.00^a^	41.67^b^

## References

[b1] Tropek R., Cerna I., Straka J., Cizek O. & Konvicka M. Is coal combustion the last chance for vanishing insects of inland drift sand dunes in Europe? Biol Conserv 162, 60–64 (2013).

[b2] Foster B. L. & Tilman D. Dynamic and static views of succession: testing the descriptive power of the chronosequence approach. Plant Ecol 146, 1–10 (2000).

[b3] Jones M. L. M., Sowerby A., Williams D. L. & Jones R. E. Factors controlling soil development in sand dunes: evidence from a coastal dune soil chronosequence. Plant Soil 307, 219–234 (2008).

[b4] McLachlan A. & Brown A. C. The ecology of sandy shores (Academic Press, Burlington, MA, 2006).

[b5] McLachlan A. Ecology of coastal dune fauna. J. Arid Environ. 21, 229–243 (1991).

[b6] van der Valk A. G. Environmental factors controlling the distribution of forbs on coastal foredunes in Cape Hatteras National Seashore. Can J Bot 52, 1057–1073 (1974).

[b7] Willis A. J. Coastal sand dunes as biological systems. Proc. R. Soc. Edinburgh 96B, 17–36 (1989).

[b8] Wilson J. B. & Sykes M. T. Is zonation on coastal sand dunes determined primarily by sand burial or by salt spray? A test in New Zealand dunes. Ecol Lett 2, 233–236 (1999).

[b9] Heykena A. Vegetationstypen der Küstendünen an der östlichen und südlichen Nordsee. In: Raabe E. W. (Ed.) Mitt. d. AG f. Floristik in Schl.-Holst. und Hamburg, p. 13 (1965).

[b10] Kachi N. & Hirose T. Limiting nutrients for plant growth in coastal sand dune soils. J Eco 71, 937–944 (1983).

[b11] Isermann M. & Cordes H. Changes in dune vegetation on Spiekeroog (East Friesian Islands) over a 30 year period. In: Carter R. W. G., Curtis T. G. F. & Sheehy- Skeffington M. J. (Eds.) Coastal Dunes, pp. 201–209 (Balkema, Rotterdam, Brookfield, 1992).

[b12] Forster S. M. & Nicolson T. H. Aggregation of sand from a maritime embryo sand dune by microorganisms and higher plants. Soil Biol Biochem 13, 199–203 (1981).

[b13] Rajaniemi T. K. & Allison V. J. Abiotic conditions and plant cover differentially affect microbial biomass and community composition on dune gradients. Soil Biol Biochem 41, 102–109 (2009).

[b14] Forster S. M. & Nicolson T. H. Microbial aggregation of sand in a maritime dune succession. Soil Biol Biochem 13, 205–208 (1981).

[b15] McLachlan A., Kerley G. & Rickard C. Ecology and energetic of slacks in the Alexandria coastal dunefield. Landscape Urban Plan 34, 267–276 (1996).

[b16] Nicolson T. H. Mycorrhiza in the Gramineae. II. Development in different habitats, particularly sand dunes. Trans Brit Mycol Soc 43, 132–145 (1960).

[b17] Rose S. L. Above and belowground community development in a maritime sand dune ecosystem. Plant Soil 109, 215–226 (1988).

[b18] Forster S. M. Microbial aggregation of sand m an embryo dune system. Soil Biol Biochem 11, 537–543 (1979).

[b19] Maun M. A. The biology of coastal sand dunes (Oxford University Press. NY, NY, 2009).

[b20] Chang E. H., Chen C. T., Chen T. H. & Chiu C. Y. Soil microbial communities and activities in sand dunes of subtropical coastal forests. App Soil Ecol 49, 256–262 (2011).

[b21] Yu J. & Steinberger Y. Vertical distribution of microbial community functionality under the canopies of Zygophyllum dumosum and Hammada scoparia in the Negev Desert, Israel. Microb Ecol 62, 218–227 (2011). 21442252 10.1007/s00248-011-9846-3

[b22] Schulz K., Mikhailyuk T., Dreßler M., Leinweber P. & Karsten U. Biological Soil Crusts from Coastal Dunes at the Baltic Sea: Cyanobacterial and Algal Biodiversity and Related Soil Properties. Microb Ecol 71, 178–193 (2016). 26507846 10.1007/s00248-015-0691-7

[b23] Van Dorst J.. Community fingerprinting in a sequencing world. FEMS Microbiol Ecol 89, 316 (2014). 24580036 10.1111/1574-6941.12308

[b24] Brockett B. F., Prescott C. E. & Grayston S. J. Soil moisture is the major factor influencing microbial community structure and enzyme activities across seven biogeoclimatic zones in western Canada. Soil Biol Biochem 44, 9–20 (2012).

[b25] Manzoni S., Schimel J. P. & Porporato A. Responses of soil microbial communities to water stress: results from a meta-analysis. Ecol 93, 930–938 (2012). 10.1890/11-0026.1 22690643

[b26] Bell C., McIntyre N., Cox S., Tissue D. & Zak J. Soil Microbial Responses to Temporal Variations of Moisture and Temperature in a Chihuahuan Desert Grassland. Microb Ecol 56, 153–167 (2008). 18246293 10.1007/s00248-007-9333-z

[b27] Chen T. H., Chiu C. Y. & Tian G. Seasonal dynamics of soil microbial biomass in coastal sand dune forest. Pedobiologia 49, 645–653 (2005).

[b28] Levin N., Kidron G. J. & Ben-Dor E. The spatial and temporal variability of sand erosion across a stabilizing coastal dune field. Sedimentology 53, 697–715 (2006).

[b29] De Rooij-van der Goes P. C. E. M., Van Dijk C., Van der Putten W. H. & Jungerius P. D. Effects of sand movement by wind on nematodes and soil-borne fungi in coastal fore dunes. J Coast Conserv 3, 133–142 (1997).

[b30] Danin A. & Yaalon D. H. Silt plus clay sedimentation and decalcification during plant succession in sands of Mediterranean Coastal Plain. Isr J Earth-Sci 31, 101–109 (1982).

[b31] Sarig S., Fliessbach A. & Steinberger Y. Soil microbial biomass under the canopy of coastal sand dune shrubs. Arid Soil Res Rehabil 13, 75–80 (1999).

[b32] Grayston S. J. & Prescott C. E. Microbial communities in forest floors under four tree species in coastal British Columbia. Soil Biol Biochem 37, 1157–1167 (2005).

[b33] Rowell D. L. Soil science: methods and applications (Longman Group UK Ltd., London, 1994).

[b34] Lane D. J. 16S/23S rRNA sequencing. In: Stackebrandt E., Goodfellow M. (Eds) Nucleic acid techniques in bacterial systematics. pp. 115–175 (Wiley, Chichester, 1991).

[b35] Nübel U. . Sequence heterogeneities of genes encoding 16S rRNAs in *Paenibacillus polymyxa* detected by temperature gradient gel electrophoresis. J Bacteriol 178, 5636–5643 (1996). 8824607 10.1128/jb.178.19.5636-5643.1996 PMC178401

[b36] Culman S., Bukowski R., Gauch H., Cadillo-Quiroz H. & Buckley D. T-REX: software for the processing and analysis of T-RFLP data. BMC Bioinformatics 10, 171 (2009). 19500385 10.1186/1471-2105-10-171 PMC2702334

[b37] Legendre P. & Gallagher E. D. Ecologically meaningful transformations for ordination of species data. Oecologia 129, 271–280 (2001). 10.1007/s004420100716 28547606

[b38] Heberle H., Meirelles G. V., da Silva F. R., Telles G. P. & Minghim R. InteractiVenn: a web-based tool for the analysis of sets through Venn diagrams. BMC Bioinformatics 16, 169 (2015). 25994840 10.1186/s12859-015-0611-3 PMC4455604

